# Nonclosure of the Peritoneum during Appendectomy May Cause Less Postoperative Pain: A Randomized, Double-Blind Study

**DOI:** 10.1155/2019/9392780

**Published:** 2019-05-23

**Authors:** Huseyin Kazim Bektasoglu, Mustafa Hasbahceci, Samet Yigman, Erkan Yardimci, Enver Kunduz, Fatma Umit Malya

**Affiliations:** ^1^Department of General Surgery, Faculty of Medicine, Bezmialem Vakif University, Istanbul 34093, Turkey; ^2^Department of General Surgery, Medical Park Fatih Hospital, Istanbul 34080, Turkey

## Abstract

**Objective:**

We aim to evaluate the effect of peritoneal closure on postoperative pain and life quality associated with open appendectomy operations.

**Methods:**

This is a single-center, prospective, randomized, and double-blinded study. Here, 18–65-year-old patients who underwent open appendectomy for acute appendicitis were included. Demographic data of the patients, operation time, length of hospital stay, pain scores using a 10 cm visual analogue scale (VAS) on the first postoperative day, quality of life assessment using the EuroQol-5D-5L questionnaire on postoperative 10th day, deep wound dehiscence, bowel obstruction, and mortality data were recorded.

**Results:**

In total, 112 patients were included in the study. The demographic data showed no significant difference between the groups. The median VAS score was lower in the group with open peritoneum, but this difference was not statistically significant (3 vs. 4, *p*=0.134). The duration of surgery was significantly shorter in the peritoneal nonclosure group (31.0 ± 15.1 vs. 38.5 ± 17.5 minutes, *p*=0.016). Overall complication rates and life quality test (EuroQol-5D-5L) results were similar between groups.

**Conclusion:**

Nonclosure of the peritoneum seems to shorten the duration of surgery without increasing complications during open appendectomy. Postoperative pain and life quality measures were not affected by nonclosure of the peritoneum. This trial is registered with NCT02803463.

## 1. Introduction

Acute appendicitis is the most common surgical emergency in children and adults [1, 2]. Appendectomy, the most prominent method in the treatment of appendicitis, can be performed with an open method or laparoscopically. Although open appendectomy was standard treatment for appendicitis in past, laparoscopic appendectomy has become popular as a treatment modality in the last decade [3]. Closure of the peritoneum is not usually preferred during laparoscopic appendectomy. However, in open appendectomy, some surgeons pay special attention to closure of the peritoneum.

According to the literature, the debate over peritoneal closure during the abdominal operations goes back to the 1930s [4]. Many studies that analyze the results of closure or nonclosure of the peritoneum during the repair of anterior wall of the abdomen evaluated gynecological and obstetric operations with regard to the impact of peritoneal closure on postoperative outcomes, such as pain scores, duration of hospital stay, postoperative complications, and adhesion formation [5–12]. The positive impact of nonclosure of the peritoneum on postoperative pain during appendectomy has been shown in only one study. Specifically, it was demonstrated that the mean visual analogue scale (VAS) score for pain was more favorable for the nonclosure group on the postoperative first day (*p* < 0.05) [13]. However, we could not find any report regarding the effect of peritoneal closure or nonclosure on the quality of life in the postoperative period. Therefore, it may be logical to expect that the peritoneal nonclosure approach after open appendectomy causes less postoperative pain and is associated with an increase in quality of life.

The purpose of this study is to compare the results of peritoneal closure or nonclosure during open appendectomy operations in terms of postoperative outcomes, pain, and quality of life.

## 2. Materials and Methods

This study was planned as single-center, prospective, randomized, and double-blind study and approved by the Local Human Ethics Committee. All of the protocols conformed to the ethical guidelines of the 1975 Helsinki Declaration, and written informed consent was obtained from all subjects. This study was registered at www.clinicaltrials.gov (NCT02803463).

The study population consists of patients aged between 18 and 65 years who were scheduled for an emergency appendectomy with a preoperative diagnosis of acute appendicitis between June 2016 and December 2016. Diagnosis and treatment of all patients were performed at Bezmialem University Faculty of Medicine Department of General Surgery. The diagnosis was confirmed with abdominal ultrasound or abdominal tomography preoperatively. Exclusion criteria include presence of intra-abdominal abscess in preoperative imaging; presence of local or diffuse purulent fluid in the abdomen during operation; pregnancy; history of malignancy, chronic liver disease, chronic renal failure, diabetes, known psychiatric, or mental disorder; and refusal of the patient to participate in the study. Patients who met these criteria were enumerated and randomized as the peritoneal nonclosure (Group 1) and peritoneal closure (Group 2) groups by computerized randomization with an allocation number of 1 for each group. Patients in the first group included those who had left the peritoneum open and had direct muscle and fascia sutures during closure of the appendectomy incision. Patients in the second group included those in whom the peritoneum is closed separately during closure of the appendectomy incision.

All patients were treated with 750 mg cefuroxime axetil via the intravenous route (Cefax, Deva, Turkey) before the operation. Open appendectomy was performed in all patients in accordance with standard appendectomy technique with a 3-4 cm Mc Burney incision. The attending surgeon was informed of the group of the patient immediately before the operation from a computer-based randomization system in the operating room. In patients with closed peritoneum, the peritoneum was sutured with continuous polyglactin suture (2/0 Coated vycrl, Ethicon, USA), the muscle layer was sutured with interrupted polyglactin sutures (2/0 Coated vycrl, Ethicon, USA), the fascia was sutured with continuous polyglactin suture (Coated vycrl, Ethicon, USA), and the skin was sutured with the interrupted polypropylene sutures (3/0 Prolene, Ethicon, USA). In the nonclosure group, the muscle layer, the fascia, and the skin were sutured in the same manner as described for the patients in the peritoneal closure group.

The appendectomy materials were routinely sent for histopathological examination, and all patients were postoperatively followed up in the general surgery clinic. Intramuscular 75 mg diclofenac sodium twice a day (Diclomec, Abdi Ibrahim, Turkey) and intravenous 750 mg cefuroxime axetil twice a day (Cefaks, Deva, Turkey) were administered postoperatively and routinely in the first 24 hours, and patients were discharged on the first postoperative day if there was no problem. During discharge, all patients were prescribed 50 mg oral diclofenac sodium twice a day if required (Abdi Ibrahim, Istanbul, Turkey). The patients were followed up at the outpatient clinic for 7–10 days during the first postoperative month.

Demographic data of the patients, operation time, length of hospital stay, pain scores using a 10 cm visual analogue scale (VAS) on the first postoperative day, quality of life assessment using the EuroQol-5D-5L questionnaire developed by EuroQol Group on the postoperative 10th day after discharge, deep wound dehiscence, bowel obstruction, and mortality data were recorded. The primary outcomes of this study were postoperative pain scores and life quality scores between the groups. Postoperative pain scores were evaluated using VAS on the first postoperative day with a range from “0” representing no pain to “10” expressing an unbearable pain. Quality of life assessment via the EuroQol-5D-5L questionnaire was performed on the postoperative 10th day with higher scores representing better health status. All VAS score and quality of life assessments were performed by clinical nurses who were blind to the groups.

### 2.1. Statistical Analysis

Sample size and power calculations were based on the mean VAS scores published previously for both peritoneal closure (4.0 ± 0.6) and nonclosure groups (3.7 ± 0.6) after open appendectomy [13]. Using these numbers, a power of 0.80, and a significance level of 0.05, 63 patients in each group were needed to demonstrate at least a 10% decrease in the mean VAS scores. Considering a 20% drop out rate, a total of 151 patients were planned to be included in the study.

Statistical evaluation was performed using the IBM SPSS Statistics for Windows, Version 21 (IBM Corp., Armonk, N.Y., USA). The comparison of independent normally distributed numerical data was performed using Student's *t*-test. The comparison of independent and nonnormal distributed data was performed using the Mann–Whitney *U* test. The comparison of ordinal data was performed using the Mann–Whitney *U* test. The comparison of nominal data was performed with Chi-square and Fisher's exact test. The results were evaluated at a significance level of *p* < 0.05 with 95% confidence intervals.

## 3. Results

**Figure 1:**
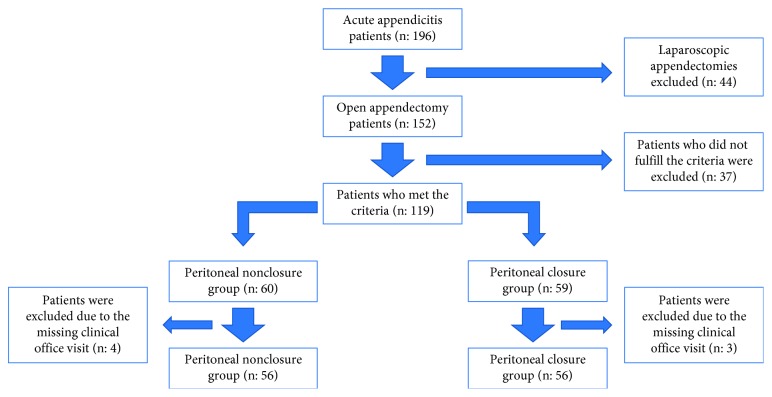
Flow diagram of patients enrolled in the study.

A total of 152 patients who underwent appendectomy were included. After exclusion of 37 patients (Figure 1), 119 patients were included in the study. Four and three patients in Groups 1 and 2, respectively, were excluded due to missing official visits. Therefore, a total of 112 patients remained for the final analysis. The mean age of these patients was 30.9 ± 12.7 years, and the female to male ratio was 39/73. The flow diagram according to the enrollment and follow-up of the patients is shown in Figure 1.

Demographic data, i.e., age, gender, and BMI, showed no significant difference between the groups (*p*=0.381,  *p*=0.843, *p*=0.619, respectively). Regarding surgery duration, in patients whose peritoneum was not closed, the duration was significantly shorter (31.0 ± 15.1 minutes) than those whose peritoneum was closed (38.5 ± 17.5) (*p*=0.016). All patients in both groups were discharged on the first postoperative day. When postoperative complications were compared, no significant difference was noted between the groups (*p*=0.728). In addition, no intra-abdominal abscess, deep wound dehiscence, or postoperative bowel obstruction occurred in any group. No mortality was observed during the follow-up period. Details are provided in Table 1.

**Figure 2:**
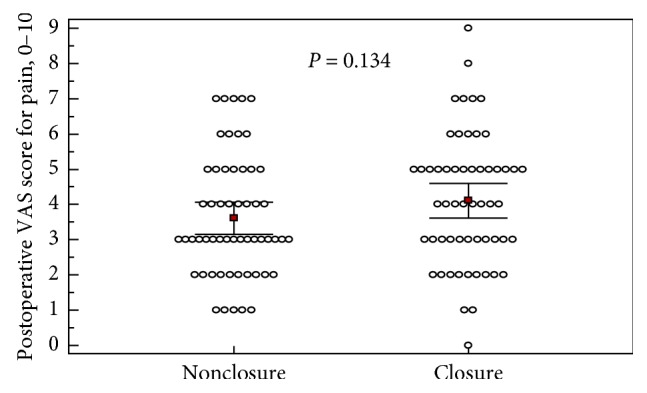
Postoperative VAS scores for pain. VAS scores for pain at postoperative day 1 are also shown. Each group includes 56 patients. Square plots show mean values (3.6 vs. 4.1) and horizontal lines show confidence intervals (3.16–4.04 vs. 3.6–4.6) at 95% level. *p* value calculated using a Mann–Whitney *U* test.

On postoperative day one, the median VAS score was lower in the open peritoneum group, but this result was not statistically significant (3 vs. 4, *p*=0.134). The post hoc power analysis for VAS score was calculated as 31.1%. Mean and confidence interval results of the groups are shown in Figure 2.

**Figure 3:**
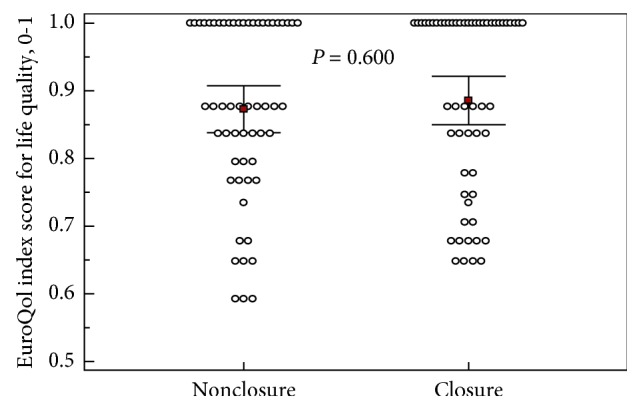
EuroQol-5D5L index scores for life quality assessment. EuroQol-5D-5L index scores for life quality assessment at postoperative day 10 are also shown. The greater value indicates better health status. Each group includes 56 patients. Square plots show mean values (0.87 vs. 0.89) and horizontal lines show confidence intervals (0.836–0.904 vs. 0.856–0.924) at 95% level. *p* value calculated using a Student's *t*-test.

**Figure 4:**
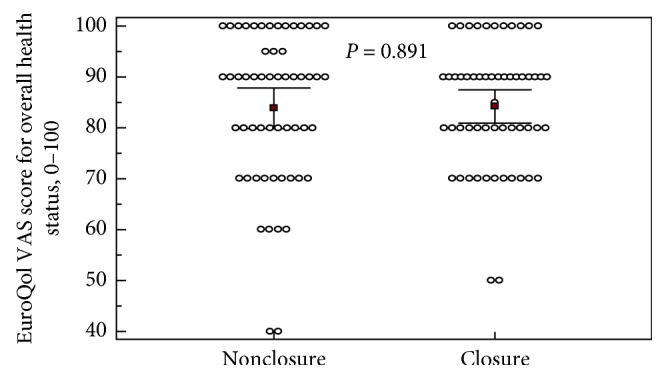
EuroQol-5D-5L VAS scores for overall health status assessment. EuroQol-5D-5L VAS scores for overall health status assessment by the patient at postoperative day 10 are also shown. The greater value indicates better health status. Each group includes 56 patients. Square plots show mean values (83.84 vs. 84.2) and horizontal lines show confidence intervals (79.9–87.8 vs. 81–87.4) at 95% level. *p* value calculated using a Student's *t*-test.

Regarding EuroQol-5D-5L results, no difference in terms of index score was noted between the two groups (*p*=0.600). No statistically significant differences in terms of visual analogue scale evaluating patient's self-rated overall health condition (*p*=0.891) were noted between the two groups. Details on the life quality tests are given in Figures 3 and 4.

## 4. Discussion

The results show that leaving the peritoneum open seems to reduce the operation time. Although it does not reach statistical significance, there is also a decrease in postoperative VAS scores for pain. In the quality of life parameters, no significant difference is found between the intergroup index scoring and the self-evaluation of overall health status via VAS. In addition, nonclosure of the peritoneum does not increase perioperative complications.

Although appendicitis is the most common surgical emergency and the treatment is generally standardized, surgeons may still use different surgical techniques [3]. One of these surgical technique differences involves closure of the peritoneum during the closure of the incision in open appendectomies.

The first study on the closure of the peritoneum in the literature was published in 1939 by Warren [4], who discussed the necessity of closing the peritoneum during appendectomy. In this study, whether leaving the peritoneum open will help the drainage of perforated appendicitis cases was assessed in this study. It was reported that it could be safe to close the peritoneum in cases of perforated appendicitis without abscess formation [4]. Despite the fact that approximately 80 years have elapsed from this study, there is still no consensus about the closure of peritoneum in laparotomies due to different concerns.

Studies evaluating closure of the peritoneum are usually performed in obstetric cases, and the adhesion criterion is in the foreground [6, 11, 12]. The probable cause is that it can also be used at births after caesarean section and provide a chance for a second look after the operation. In a prospective randomized study [11] evaluating the effect of peritoneal closure on adhesions, patients were divided into two groups according to whether the visceral and parietal peritoneum were closed together, and peritoneal adhesion was assessed in patients with secondary cesarean operations via a score sheet by the attending surgeon. There was no difference in the development of adhesions between two groups. Leon et al. [14] conducted a study on dogs to evaluate the efficacy of closing the peritoneal layers during laparotomy, and no difference in the development of adhesions was noted between the cases where the peritoneum was closed and not closed.

Khan et al. [15] reported that adhesions and small bowel obstruction develop due to the suture material used during robotic inguinal hernia. Another case report mentioned adhesion and small bowel obstruction due to self-anchoring barbed suture used after transabdominal preperitoneal hernioplasty [16]. In our study, patients were not evaluated for intra-abdominal adhesions because none of the patients underwent a second abdominal operation for another reason during the follow-up period, and none of these patients developed clinically relevant bowel obstruction.

Operation time, the length of hospital stay, and postoperative pain regarding the closure of peritoneum have been evaluated in many studies, and most of these studies involved gynecological and obstetric operations [5, 8, 10]. In a prospective randomized study by Kurek Eken et al. [5], 128 patients with cesarean section were divided into four equal groups. VAS score for pain, operation time, postoperative complications, and duration of hospital stay were evaluated among these four groups, including closure of only the parietal peritoneum, closure of only the visceral peritoneum, closure of the visceral and parietal peritoneum, and no closure of the peritoneum. In the group in which visceral and parietal peritoneum were closed, the operation time was significantly longer, and the VAS score was higher than that of the other groups. In a prospective study evaluating the operation time and the pain score in 100 patients after cesarean section, the pain score was lower and the operation time was shorter in the group in which the peritoneum was not closed [8]. In our study, it was observed that closure of the peritoneum had no effect on postoperative early complications, but nonclosure of the peritoneum appeared to reduce the operation time. In a review by Gurusamy et al. [9], five prospective randomized trials comparing closure or nonclosure of the peritoneum in nonobstetric operations were analyzed. The peritoneum was closed in 410 of 836 patients, and the peritoneum was not closed in 426 cases. Catgut or chromic catgut was used in four of the five studies for the closure of peritoneum. Incisions in the studies show heterogeneity with three vertical and two transverse incisions. Perioperative burst abdomen was assessed in three of these trials, and there was no difference between the groups in terms of the overall abdominal burst. In addition, incisional hernia rates were examined, and no difference in overall incisional hernia rates was observed. Only one of these studies evaluated the length of hospitalization, and no difference between the groups was observed. This review revealed that closure of the peritoneum has no short- or long-term advantages in nonobstetric cases. Moreover, none of the studies evaluated in review reported any data with regard to the patients' quality of life, intestinal obstruction rates, and reoperation rates due to incisional hernia or adhesions [9, 13, 17–20].

Suresh et al. [13] examined closure of the peritoneum in open appendectomies. In total, 100 patients who underwent open appendectomy for appendicitis were included in the study, and the patients were divided into two equal groups according to the closure status of the peritoneum. The study results demonstrate that, in the group in which the peritoneum was not closed, the operation time, VAS score for pain, and the postoperative analgesic requirement were significantly reduced compared with the group in which the peritoneum was closed. In our study, the postoperative pain score was lower in the open peritoneum group when the median values were compared. However, this difference did not reach statistical significance. The calculated post hoc power remains lower than expected. Therefore, the increased number of the patients may help to obtain a significant finding on postoperative pain scores for further studies. The effect of the peritoneal closure on quality of life was evaluated using the EuroQol-5D-5L test in the present study. The Euroqol-5D-5L test is a standardized questionnaire to assess the overall health status of the patients and is widely in use [21–24]. Calculation of the index score for this test was based on United Kingdom values since there is no verified value for our country. Self-assessment of the general health condition of the patients as a variable of the test was performed with a visual analogue scale of 20 cm and a range of 0–100 points, and no significant difference was found between the two groups. Although nonclosure of the peritoneum positively affected the pain scores on the postoperative 1st day, there was no difference in the scores on the 10th postoperative day.

The limitations of this study are as follows. The incision length was not evaluated due to the lack of information on the proportion of the incision length to the abdominal morphology. The difference in the postoperative VAS scores between groups was not statistically significant due to the probable lack of power given that laparoscopy has become popular during appendectomy [3, 25]. In addition, the life quality test results were not compared with the literature due to the use of the EuroQol-5D-5L test.

## 5. Conclusion

Leaving the peritoneum open seems to reduce the operation time. Although it does not reach statistical significance, there is also a decrease in postoperative VAS scores for pain. Regarding the quality of life parameters, no significant difference between the intergroup index scoring and the self-evaluation of overall health status is noted via VAS. Nonclosure of the peritoneum does not increase perioperative complications. Decreased postoperative VAS scores may guide nonclosure of the peritoneum in other incisions used in laparotomy.

## Figures and Tables

**Table 1:** Demographic data, operation time, VAS scores for postoperative pain, and postoperative complication details of the patients.

	Group 1 (open)	Group 2 (closed)	*p* value
Age (years)	31.8 ± 10.0	30.1 ± 10.4	0.381

Gender			
Female	19	20	0.843
Male	37	36	

BMI (mean ± SD)	24.5 ± 3.4	24.8 ± 2.4	0.619

Operation time (minutes) (mean ± SD)	31.0 ± 15.1	38.5 ± 17.5	0.016

Superficial wound infection			
Yes	4	3	1.000
No	52	53	

Deep wound infection			
Yes	1	1	1.000
No	55	55	

Overall complication			
Yes	5	4	0.728
No	51	52	

## Data Availability

The data used to support the findings of this study are available from the corresponding author upon request.
